# Quantification of dissolved O_2_ in bulk aqueous solutions and porous media using NMR relaxometry

**DOI:** 10.1038/s41598-020-79441-5

**Published:** 2021-01-11

**Authors:** Kurt Livo, Manika Prasad, Trent R. Graham

**Affiliations:** 1 Center for Rock & Fluid Multiphysics, Colorado School of Mines, Golden, CO 80401 USA; 2 Pacific Northwest National Laboratory, Richland, WA 99352 USA

**Keywords:** Experimental nuclear physics, Marine chemistry, Geophysics, Freshwater ecology

## Abstract

Effects of dissolved paramagnetic oxygen (O_2_) in water on ^1^H nuclear magnetic resonance (NMR) Carr-Purcell-Meiboom-Gill (CPMG) experiments is evaluated at a ^1^H Larmor frequency of 2 MHz. Dissolution of O_2_ into water significantly reduces the ^1^H transverse relaxation coefficient (T_2_). For deoxygenated water, T_2_ is 3388 ms, water at ambient atmospheric conditions (7.4 mg/L O_2_) exhibits a T_2_ of 2465 ms, and dissolution of 2710 mg/L O_2_ further reduces T_2_ to 36 ms. The results were fit with an empirical model to facilitate prediction of T_2_ times for bulk water as a function of paramagnetic oxygen concentrations in solution. Dissolved O_2_ also greatly influences ^1^H NMR CPMG experiments of confined water in a model system composed of Berea sandstone. For this system, 90 mg/L O_2_ in H_2_O enhances T_2_ relaxation of bulk water such that the relaxation time is comparable to physically confined water in the sandstone pores. Given the sensitivity of NMR T_2_ coefficients to paramagnetic oxygen, low-field NMR-based characterization of fluid and porous media structure requires control of dissolved oxygen, as geospatial variation in the partial pressure of O_2_ alone is expected to perturb fluid and pore relaxation times by up to 60 and 36%, respectively.

## Introduction

Low-field (2 MHz) nuclear magnetic resonance (LF-NMR) relaxometry provides physical properties of hydrogen-bearing fluids such as viscosity. LF-NMR experiments of porous media saturated with hydrogen proton bearing fluids yield pore structure information such as porosity, pore size distribution, and permeability^1–3^ by probing interactions of fluids with grain surfaces^4–7^. In such porous media studies, surface relaxation mechanisms, primarily controlled by paramagnetic elements such as iron and manganese associated with clay minerals and sulfides on grain surfaces, yield faster relaxation times^8–12^. The paramagnetic surface properties of the grains dominate proton relaxation at time scales greatly below bulk relaxation or diffusion-driven mechanisms leading to a simplified NMR relaxation equation that ignores free fluid and diffusion responses.

Qualitative NMR studies suggest that the presence of oxygen molecules in solution alters both electron spin and nuclear spin relaxation rates^10,13–15^. In high-field NMR spectroscopy, enhanced relaxation due to paramagnetic oxygen in proteins and lipids at elevated pressures yields information about protein structure and interactions^16–20^. At low magnetic fields and in simple aqueous solutions of deoxygenated water, the experimental ^1^H transverse relaxation coefficient (T_2_) is between 3400 and 3600 ms^21,22^. In LF-NMR studies, dissolved oxygen in aqueous solutions disrupts proton precession and decreases relaxation times^15,22–24^. In addition to influencing nuclear spin relaxation in aqueous systems, dissolved oxygen in organic solutions has been found to reduce the relaxation time of hydrocarbons with dependence on hydrocarbon molecular weight, correlating with the dependence on the oil’s density and viscosity^25–27^. A predictive understanding of the effects of dissolved oxygen is similarly fundamental to the calculation of fluid properties and porous media structure.

To evaluate the sensitivity of dissolved oxygen on LF-NMR T_2_ based characterization of fluid properties and porous media structure, T_2_ relaxation times in bulk water were determined as a function of paramagnetic oxygen. An empirical formula was then developed to relate the dissolved oxygen concentration to the T_2_ of bulk water. In addition, the effects of dissolved O_2_ were demonstrated in water saturated sandstone cores. The results demonstrate that dissolved paramagnetic O_2_ in water saturated sandstone cores alters the LF-NMR T_2_ relaxation times of water by two orders of magnitude to overlap with T_2_ relaxation times typically attributed to water interacting with the surface. This work demonstrates that dilute amounts of dissolved oxygen produces significant errors in calculation of pore size distributions and permeability, and that characterization of fluid properties and porous media structure is best done under anoxic conditions.

## Results

### Studies of dissolved oxygen in bulk water

**Figure 1:**
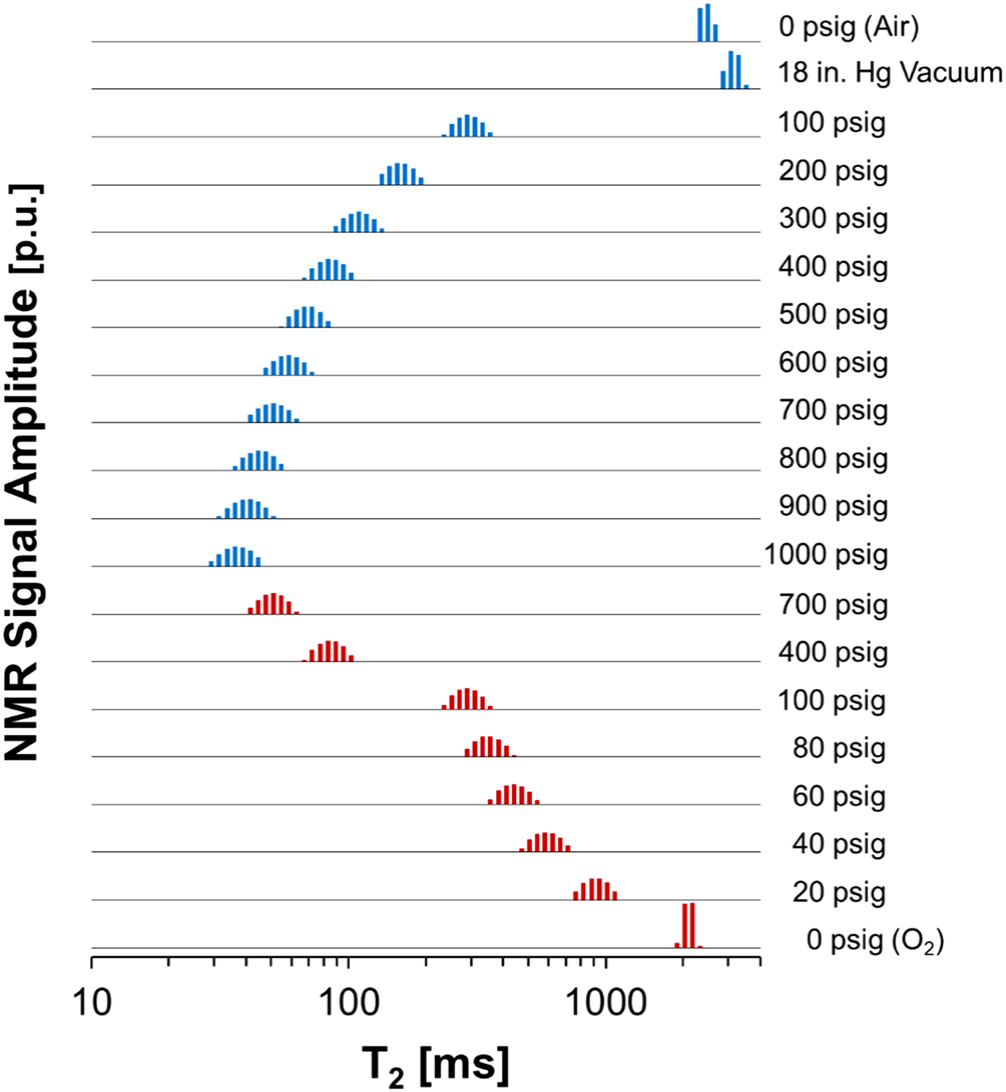
^1^H NMR T_2_ relaxation distributions of bulk H_2_O during O_2_ pressurization (blue) and depressurization (red). Distributions are shifted vertically to prevent overlap in order to show relative changes at each pressure step. The distributions sum to unity, with additional discussion of y-axis intensities detailed in the main text.

The initial results detail the effects of dissolved oxygen on the T_2_ properties of bulk water. The T_2_ logarithmic mean (T2LM) of the distributions inverted from the NMR magnetization decay were found to decrease systematically with pressure as shown in Fig. 1. Additional details, such as the mole fraction of O_2_ in solution, are provided in Table S1 in the *Supporting Information*.

To provide a baseline T_2_ value for water at laboratory pressure and temperature (PT) conditions, T_2_ was measured for settled DI water in a pressure vessel under ambient atmosphere and ambient PT. As shown in Fig. 1 and Table S1, the T2LM value of H_2_O at laboratory PT conditions (pO_2_ = 2.50 psia, where pO_2_ is the partial pressure of oxygen) was 2465 ms. With application of vacuum, T2LM increased to 3129 ms at pO_2_ = 0.64 psia (vacuum of − 18.0 in. Hg), and to 3388 ms at pO_2_ = 0.02 psia (− 24 in. Hg vacuum). Then by pressurization with 100 psig O_2_ gas, the T2LM of H_2_O decreased 91% to 294 ms, relative to the vacuumed state (− 18.0 in. Hg). Further O_2_ pressurization up to 1000 psig yielded a final T2LM value of 37 ms.

The decrease in the breadth of the T2LM distribution at higher oxygen pressures can be attributed to the logarithmic spacing of the inversion using the Lawson and Hanson method^28^ where more data sampling occurs at smaller time intervals. This causes the bin size of the T_2_ distribution to vary nonlinearly. The higher amount of sampling steps at smaller time intervals leads to greater precision in T2LM calculations at small T_2_, which can be seen by the narrower distributions at the higher oxygen pressures.

**Figure 2:**
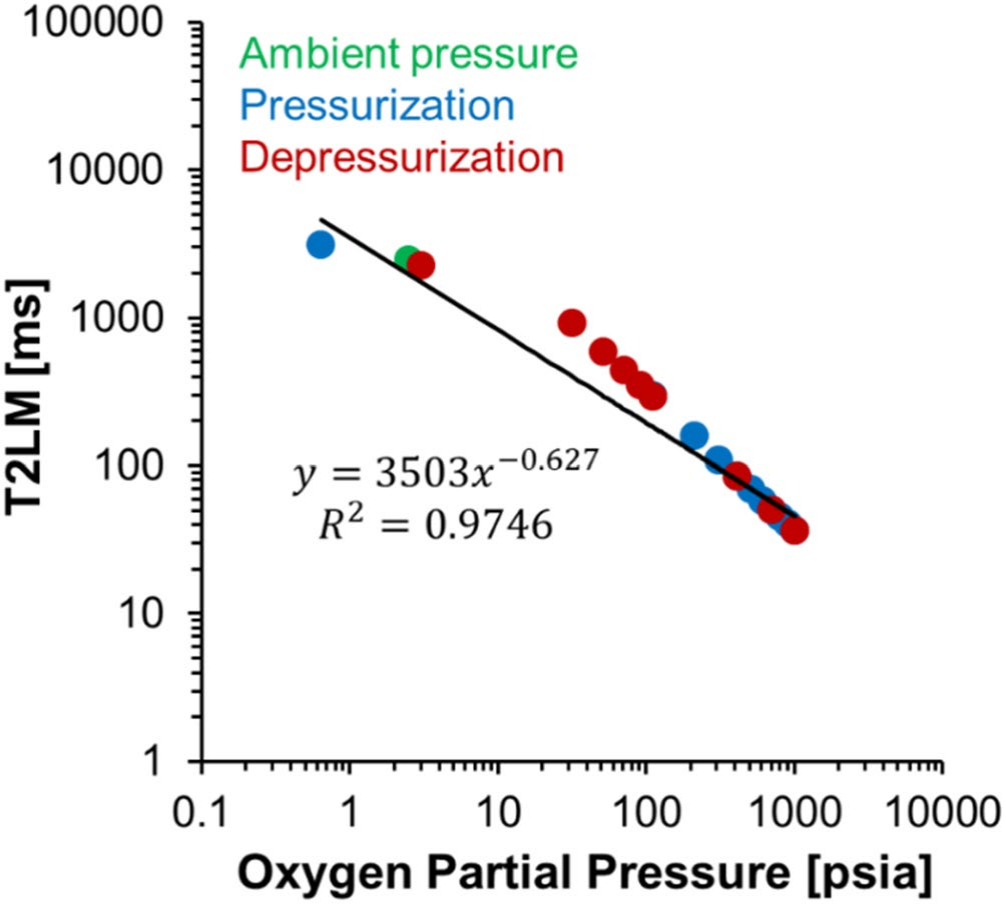
T2LM values of bulk H_2_O as a function of O_2_ pressure shown on a log–log scale. Note the large reduction in relaxation of the fluid within the first 100 psi. Good agreement is seen in the T2LM values at 700, 400, and 100 psig regardless of the pressure path. The empirical correlation presented utilizes only pressurization data points following vacuuming of the sample. The line is the regressed power law function, which corresponds to an R^2^ value of 0.9746.

Repeated pressure steps in Fig. 2 demonstrate overall agreement between T2LM values during pressurization and depressurization with oxygen. For example, the differences in T2LM are less than 4 ms at pressures greater than 100 psig. However, there is imperfect hysteresis upon return to 0 psig. After depressurization and subsequent vacuum to − 18.0 in. Hg to match the pressure of the initial state, the T2LM value was 2238 ms, more than 890 ms shorter than the initial value of 3129 ms. The deviation between T2LM values at ambient pressure is attributed to the presence of a mixture of dissolved gases from air, and is further detailed in “Discussion” section.

The systematic reduction in T2LM with absolute O_2_ partial pressure is well fit by a power law (Fig. 2). The power law model to describe the T2LM – pressure relationship at 21.1 °C, is:

1 $$ {\text{T2LM }} = { 35}0{3}*{\text{pO}}_{{2}}^{{ - 0.{627}}} $$

where, T2LM is T_2_ logarithmic mean with units of ms and pO_2_ is the absolute oxygen partial pressure with units of psia.

### Studies of dissolved oxygen in water-saturated Berea sandstone

Next, the effect of pO_2_ variation on T_2_ relaxation was evaluated for a water-saturated Berea sample with excess free-fluid. The bulk fluid surrounding the core prevents drying at varying oxygen pressures and comprised 91.46% of the total detectible water fraction in the pressure vessel at ambient conditions. Due to the multi-exponential decay of NMR signals, discrete portions of the T_2_ distributions can be identified for the bulk fluid, large pores, and small pores of the Berea core.

**Figure 3:**
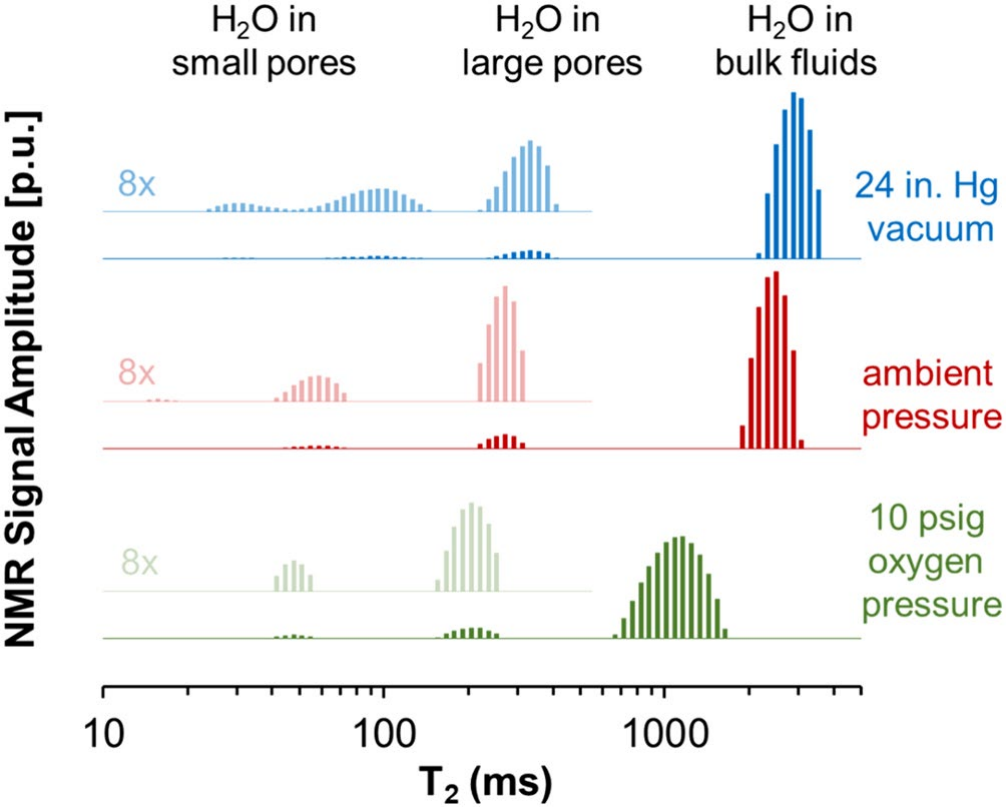
^1^H NMR T_2_ relaxation distributions of water-saturated Berea core samples with excess bulk fluid surrounding the core under vacuum, at ambient pressure, and with an applied pressure of 10 psig O_2_. A portion of the distribution is magnified ×8 and offset to facilitate inspection of the components attributed to water in large and small pores. The magnified portion is annotated and shown with greater transparency.

The bulk fluid portion resides in between the pressure vessel and core surfaces, and exhibits a smaller T_2_ than bulk water (Fig. 1) likely due to surface interactions. NMR measurements of the Berea core at ambient pressure in air (pO_2_ = 2.50 psia) resulted in a T2LM of 2407 ms for bulk water, 264 ms for water in the large diameter pores, and 53 ms for water in small diameter pores (Fig. 3). When subjected to vacuum of − 24.0 in. Hg (pO_2_ = 0.02 psia), the T2LM of the bulk fluid increased to 2872 ms. Similarly, T2LM of water in the pore fractions also increased to 317 ms in large pores and to 72 ms in small pores. As tabulated in Table S2 in the *Supporting Information*, measurements performed with 10 psig (pO_2_ = 21.90 psia) of oxygen decreased the T2LM values to values of 1086 ms for bulk fluid, 204 ms for water in large pores and 48 ms for water in small pores. Notably, in addition to changes in T2LM values, there was also a reduction in the total detectable pore fluid by about 11.3% following O_2_ pressurization.

## Discussion

Paramagnetic properties of O_2_ are known to decrease ^1^H NMR T_2_ relaxation times^14,15,23,24,29^. Mechanistically, this is caused by a disruption of the hydrogen precession by dissolved O_2_^14,22,27^ controlled by the distribution and magnetic susceptibility of paramagnetic agents in solution. The number of molecular collisions between the paramagnetic oxygen and hydrogen bearing molecules of the DI water during Brownian motion can dominate relaxation pathways even with dissolved oxygen concentrations at pressures as low as 20 psig. The oxygenated free water relaxation time can coincide with relaxation times typical of surface relaxation mechanisms in porous media.

Changes in solution composition due to pressurization with O_2_ can be estimated from Multiflash calculations where an increase in oxygen from the vacuumed state to 100 psig yields a molar fraction of oxygen in solution of only 2.00 × 10^–4^ mol O_2_/mole H_2_O. Therefore, even trace amounts of dissolved O_2_ lead to a massive 90.62% reduction in relaxation time (Figs. 1 and 2). The increase in gas in solution from atmospheric pressure to 100 psig results in an increase in oxygen concentration from 2.43 × 10^–5^ to 1.68 × 10^–4^ mol O_2_/mole H_2_O (43.25 to 297.97 mg O_2_/L H_2_O) and an order of magnitude reduction in the ^1^H NMR transverse relaxation coefficient. Oxygen pressures greater than 100 psi results in smaller changes in relaxation times because the addition of more O_2_ molecules in solution is above a critical concentration where increased collision rate yields little additional relaxation.

We can characterize the number of collisions between O_2_ and H_2_O molecules in solution due to Brownian motion using a characteristic distance traveled by an O_2_ molecule in solution during a time increment equivalent to the T_2_ relaxation coefficient. Using the diffusion coefficient ($D$) of O_2_ in water of 2 × 10^–5^ cm^2^/s^30^, the characteristic diffusion length of O_2_, $L$ [cm], is:

2 $$L=\sqrt{6*D*T2LM},$$

Using the intermolecular oxygen distances of water in a stable dimer configuration of 2.97 Å^31^, the number of total collisions between water and oxygen molecules during the timespan of T2LM ($C$) was calculated for each pressure per volume using Eq. (4):

3 $$C=(L*n)/d,$$

where $C$ is the number of O_2_ collisions with water molecules per cm^3^ of volume [cm^-3^], $L$ is the aforementioned characteristic diffusion length of O_2_ in solution during T_2_ relaxation [cm], $d$ is the intermolecular oxygen distances of water [cm], $n$ is the number of O_2_ molecules per volume of water [cm^−3^].

The total number of collisions increases from 2.35 × 10^22^/cm^3^ H_2_O at − 18.0 in. Hg vacuum (pO_2_ = 0.64 psia), to 3.60 × 10^24^/cm^3^ H_2_O at an oxygen pressure of 1000 psig. The increase in the number of molecular collisions between H_2_O and O_2_ in solution results in the reduction of NMR T2LM from 3129 to 37 ms. The reduction in relaxation time occurs with a linear increase in the solubility of oxygen. As a result, the main mechanism of surface relaxation observed in NMR studies of porous media can be masked by fluid effects in oxygenated fluid systems, even at O_2_ pressures below 100 psig.

**Figure 4:**
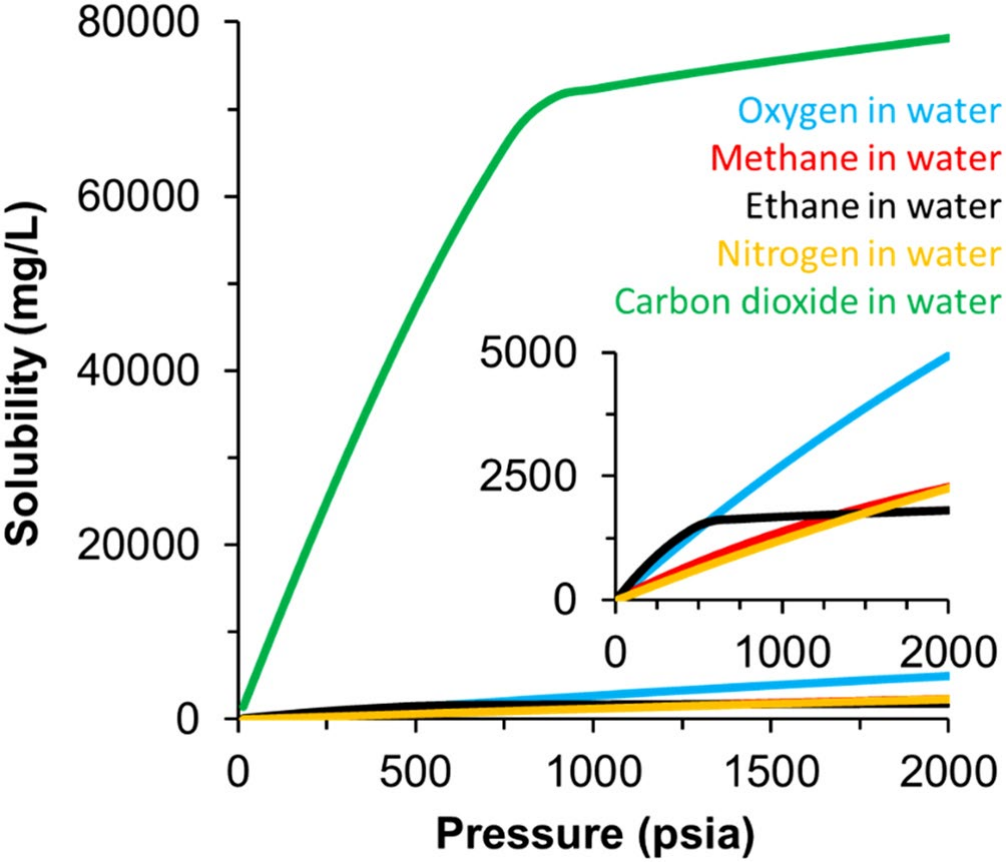
Solubility of different gases as a function of pressure in DI water generated using Multiflash advanced thermodynamic modeling software. The inset image is used to show the lower solubility values of common hydrocarbon and non-hydrocarbon gases: oxygen, methane, ethane, and nitrogen over the pressure range of the calculated values. The inflection of the solubility curves from near linear trends (Ethane and Carbon Dioxide) is caused by conversion of the gases into the liquid or critical phase at that pressure step.

The imperfect hysteresis of T2LM in bulk water following pressurization and depressurization is next discussed (Fig. 2). The imperfect hysteresis can be attributed to the greater partial pressure of oxygen in the headspace relative to that in air, which occupied the headspace in the initial measurement. In addition, other gases in air can be more soluble than O_2_. For example, Fig. 4 shows that CO_2_ in aqueous solution is approximately 30 times more soluble than O_2_ at comparable pressures. Conversely, the solubility of O_2_ is only about twice that of N_2_ and ethane and methane in solution at corresponding pressures showing that other gas fractions compete with O_2_ in solution. The vacuumed sample prior to pressurization still contains trace amounts of N_2_, O_2_, CO_2_ and other trace amounts of gases from the atmosphere. As pressurization with pure O_2_ occurs, these trace fractions are replaced with higher relative percentages of O_2_ in solution. Following the depressurization of the DI water sample, higher relative percentages of oxygen in solution account for the difference in the starting value of 3129 ms (pO_2_ = 0.64 psia) to 2238 ms (pO_2_ = 3.06 psia) values of relaxation at an applied vacuum of − 18 in. Hg.

The empirical relation presented in this work provides a correlation between NMR relaxation and O_2_ in solution as a function of pressure that could be well fit by a power model. This is observed by the linear trend of the line of best fit on the log–log plot (Fig. 2) where an increase in relative concentrations of O_2_ results in a proportional inverse relative change in relaxation time of water, a trend well described by power law relationships. Similarly, power law relationships are well established to model behaviors in fluid properties such as viscosity. Here, our power law relation describes the increased disruptions in precessional relaxation of hydrogen in solution with introduced paramagnetic O_2_ due to reduced O_2_ diffusional distance and time before proton interaction occurs, increased frequency of random molecular interaction between O_2_ and H_2_O due to Brownian motion, and increased total paramagnetism of the fluid. In this fitting equation, the power law constant of − 0.627 is defined by these multivariate interactions that result in enhanced fluid relaxation in NMR controlled by temperature, viscosity modification with introduced O_2_, and paramagnetic properties of the gas in water. Our empirical relation similarly shows a lack of well-defined T2LM values at low pressures, a limitation typical of many power law systems. However, our experimental maximum relaxation time of oxygen-free water (3.4 s), is in agreement with the Chiarotti et al. value of 3.6 s and the theoretical relaxation of water = 3.4 s from Debye’s relaxation model^22^. The high R^2^ value of 0.9746 in the experimental data shows good fit of the power law equation used to describe T2LM with an increase in O_2_ pressure across the pressure steps of our study.

**Figure 5:**
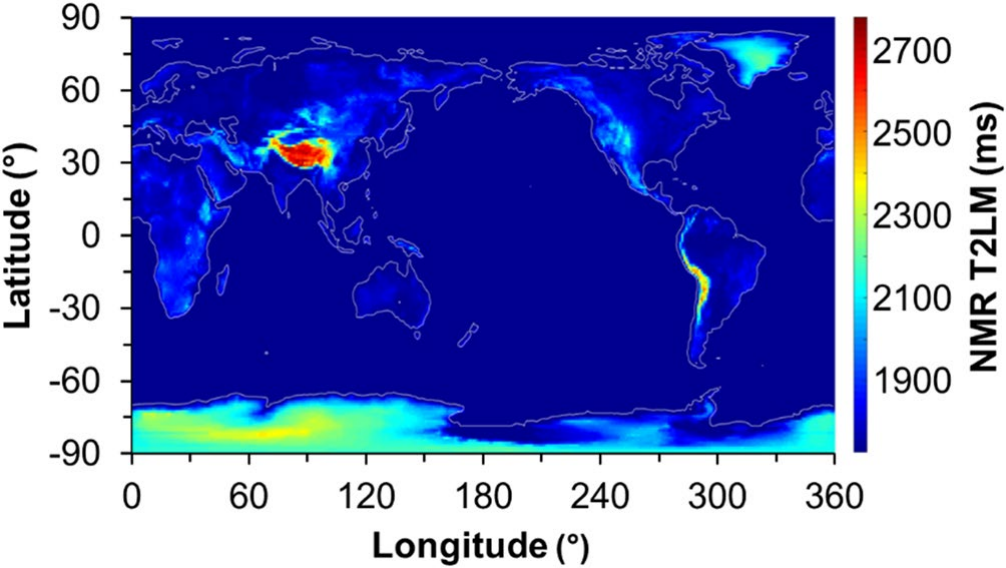
Global T2LM values calculated from topographic elevations and calculated partial oxygen pressures in 1 Degree Longitudinal and Latitudinal steps (generated using MATLAB Software Version 9.4, https://www.mathworks.com/products/compiler/matlab-runtime.html). Values range from 1728 ms at sea level to 2775 ms in the Himalayan region resulting in a 60.59% variation in water T2LM globally.

Utilizing this empirical relation, the partial pressures of oxygen in the location of this study (Golden, CO with a pO_2_ of 2.50 psi) corresponds to a T2LM value of 1973 ms for bulk H_2_O, whereas in Houston, TX (pO_2_ = 3.08 psi) the expected T2LM value would be 1732 ms. Discrepancies between these predicted values with our experimental values result from the choice of a power-law empirical model, as models with additional parameters could better fit data collected at O_2_ pressures below1 atm used. Based on the power law model and assuming isothermal conditions, values of T2LM can be estimated globally from elevation data (Fig. 5). The estimated geospatially dependent T2LM varies between 1728 ms at sea level to 2775 ms on top of the Himalayan Mountains. The partial pressure differences in oxygen in the atmosphere can cause changes in T2LM relaxation times of up to 60.59% depending on global geographic location.

Demonstration of these changes in relaxation time due to changes in oxygen concentration is also observed in porous samples where surface relaxation is commonly attributed as the main mechanism of NMR relaxation. Reduction in the partial pressure of oxygen by 2.48 psi yields a 53 ms increase in the relaxation time for large pores and a 19 ms increase in the small pores of a Berea sample going from the ambient state to vacuum state. This yields an increase in relaxation time of 19.93% in the larger pores and 36.02% for the smaller pores respectively. Similarly, pressurization of oxygen from the vacuum state (pO_2_ = 0.02 psi) to 10 psig (pO_2_ = 21.90 psi) results in a reduction of T2LM in the larger pores of 113 ms and a reduction of 24 ms in the smaller pores. This yields a reduction in relaxation times of 35.69% and 33.36% in the larger and smaller pores, respectively. Similarly, the 11.3% reduction in the detectable pore fluid signal further indicates that relaxation enhancements of the interstitial fluid result in proton relaxation times of faster environments (such as in surface dominated relaxation of small pore fractions) that are undetectable at our instrument resolution. Thus, we find that variations in pore relaxation time of sandstones up to 36% can occur with changes in O_2_ partial pressure of less than 3 psi.

## Conclusions

This work provides a quantitative relationship between NMR relaxation of water with O_2_ concentration in solution at different pressure steps, and demonstrates that T2LM can be well predicted by a power law equation. The theoretical T2LM of bulk water without the presence of oxygen is approximately 3500 ms, which is in good agreement with values found in the literature. Variations of up to 61% in T2LM of bulk water can occur depending on global location, and variations in pore relaxation of up to 36% in simple porous media can occur with changes in O_2_ partial pressure of less than 3 psi. This work demonstrates how trace impurities of paramagnetic gases in solutions cause great changes in the NMR relaxation at low magnetic fields. The impurity-altered relaxation time of pure fluids that are controlled by bulk relaxation mechanisms approach and mask surface dominated relaxation effects in porous media. We suggest that models of pressure and temperature dependent NMR response of porous media correct for oxygen content that may skew the NMR spectra due to enhanced relaxation caused by the paramagnetism of the gas. Similarly, variation of oxygen in solution with temperature, salinity, or other gases could result in appreciable differences in the NMR response that should be accounted for with our empirical relationship for more accurate predictions of pore properties with low-field NMR relaxometry.

## Experimental methods

### Experimental setup

**Figure 6:**
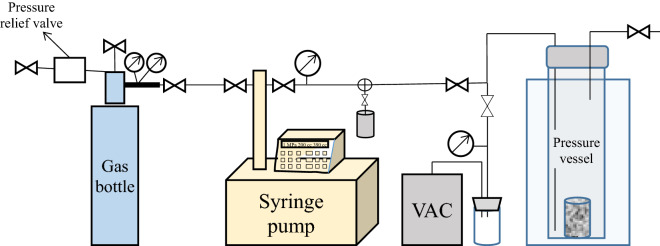
Laboratory schematic of the NMR pressure setup for pressurization of DI water samples used in this study. Gas was pressurized by charging an ISCO syringe pump from the gas cylinder and applying pressure to the pressure vessel. Valves mark shutoff values to control gas flow to the system. The NMR pressure vessel was placed into a low-field (2 MHz) Magritek NMR. A fluid trap was utilized to prevent fluid from entering the vacuum pump. The vacuum pump was used to evacuate atmospheric air from the system prior to pressurization with oxygen.

NMR Transverse (T_2_) relaxation times of DI water with dissolved research grade O_2_ was measured as a function of O_2_ pressure up to 1000 psi (about 6.9 MPa) and modeled oxygen solubility for each pressure step. NMR T_2_ relaxation was also collected in water saturated Berea sandstone cores as a function of oxygen pressure in an experimental setup consisting of a gas cylinder connected to a syringe pump, supplying gas to a pressure vessel placed in a low-field NMR (Fig. 6). The pressure vessel consisted of a machined, 2 in. outside diameter Torlon rod with a 1-in. inner diameter. The pressure vessel was sealed with a Swagelok cap with 2 fluid flow through lines: the pressure line, and a vent line opened to atmosphere. The entire pressure vessel was placed in the NMR probe. A gas cylinder was connected to the inlet of a syringe pump (ISCO Teledyne) to supply gas to the system and the syringe pump was used for pressurization of the supplied gas. A vacuum pump and fluid trap was connected to the setup via a vacuum line to evacuate the pressure vessel and pressure lines prior to introduction of gas.

### Sample preparation

DI water (Type 2 ACS Grade) was prepared from filtered tap water. Conductivity of the DI water (6.3 × 10^–5^ S/m) was determined from DC conductivity probe measurements and the total dissolved solids (TDS) content of the DI water was 28 parts per billion. The DI water was allowed to settle in a sealed container without agitation to allow maximum degassing (at laboratory pressure prior to the experiment). A known volume of this so-called settled DI water was decanted into the pressure vessel with minimal agitation to minimize any oxygenation from the atmosphere. A volume of 25.0 mL (containing approximately 2.8 g of hydrogen) ensured high signal-to-noise (SNR) measurements.

### Pressurization and depressurization

An ambient absolute pressure of 11.90 psia was derived for atmospheric pressure in Golden, Colorado at an elevation of 5675 ft (1730 m). In determination of O_2_ partial pressure from the atmosphere, air and oxygen gases are treated as ideal gas mixtures. Assuming a concentration of O_2_ in air as 21%^32^, a partial pressure of O_2_ (pO_2_) = 2.50 psi was used for calculation of oxygen pressure at ambient state. Atmospheric pressure (11.90 psia) was added to gauge pressure values for calculation of applied oxygen pressure at each pressure step. An additional study used a more efficient vacuum pump connected to the system to achieve pressures of − 24 in. Hg vacuum pressure. The value from this separate study is included here for reference to this work. Oxygen partial pressures were determined to be 0.64 psi and 0.02 psi at vacuum steps of − 18.0 in. Hg and − 24 in. Hg (where 1 psi = 2.03602 in. Hg), respectively. Henceforth in this paper, references to pressure are provided in absolute oxygen partial pressure (psia) unless otherwise noted as gauge pressures (psig).

Research grade, 99.99% pure, O_2_ (Airgas) was introduced into the pressure vessel containing the degassed water sample. O_2_ pressure was increased using the syringe pump in 100 psi increments up to 1000 psig. Depressurization steps were also controlled by the syringe pump, and the spent gas was vented to atmosphere. Pressure was decreased from 1000 to 100 psig in steps of 300 psi to document the changes in T2LM values between 100 and 1000 psig. Below 100 psig, pressure was decreased in smaller pressure steps of 20 psi for higher resolution of T2LM changes from 0 to 100 psig. Finally, a vacuum of − 18 in. Hg (pO_2_ = 3.06 psia following pressurization in a pure oxygen environment) was applied to the system. Pressure values were taken by agreement with the syringe pump and pressure gauge. Pressure was maintained with the syringe pump until the NMR readings stabilized. Gentle rocking of the pressure vessel facilitated gas entering (pressurizing) or leaving (depressurizing) the solution. Continuous fluid phases have no change in T_2_ spectra following formation of discrete bubbles of differing fluid composition^33^. Similarly, Scardina and Edwards show negligible nucleation of bubbles in water without temperature or pH changes at pressures higher than 1 atm.^34^, allowing us to ignore the effects of bubble formation in our NMR results.

### NMR experiments

NMR measurements were performed with a low-field (2 MHz) NMR (Magritek Rock Core Analyzer (RCA)) with a 2-in. probe aperture. T_2_ relaxation was acquired using Carr-Purcell-Meiboom-Gill (CPMG) pulse sequences^35,36^. The data were inverted using Laplace non-negative least square (NNLS) fitting^28,37^ for 200 logarithmically spaced time steps across the bulk relaxation time range (0.01–10,000 ms). Prospa Programming by Magritek was utilized for inversions.

The T_2_ signals were collected with a minimum SNR of 100. Sufficiently long inter-experimental delay times (1000–20,000 ms), number of echoes (2500–100,000), at an echo spacing (TE) of 100 µs were collected until full decay of the magnetic signals was observed in the NMR CPMG measurements. A background reading of the empty pressure vessel was collected at a SNR of 200 for increased measurement resolution of the background signal. This background was subtracted from each fluid measurement to ensure inverted magnetic decay trains were solely the result of the fluid response. Measurements were collected on the as-introduced sample of DI water. A vacuum of − 18.0 in. Hg was applied to the entire system by opening all valves and closing the vent valve of the pressure vessel (Fig. 6). Gently rocking the pressure vessel at discrete intervals helped remove gas from the fluid during the degassing process. NMR measurements were made at discrete time intervals between pressure steps until the relaxation time did not change—typically after a wait time of 36 h. The solution was deemed fully degassed when successive NMR measurements showed no change in the relaxation time of the sample based on T_2_ relaxation distribution shape and the logarithmic mean of the NMR transverse relaxation data (T2LM) value, given by:

4 $${\text{T}}2{\text{LM}} = \exp \left[ {\frac{{\sum\nolimits_{\text{i}} {{{\text{a}}_{\text{i}}}} \ln \left( {{{\text{T}}_{2{\text{i}}}}} \right)}}{{\sum\nolimits_{\text{i}} {{{\text{a}}_{\text{i}}}} }}} \right]$$

where, T2LM = NMR logarithmic mean of the spectra [ms], a_i_ = amplitude of spectra at inversion step i, and T_2i_ = Transverse NMR relaxation time at inversion step i [ms].

### Solubility modeling

We quantified the amount of O_2_ and other gases commonly dissolved in DI water in solution at each pressure step, by modeling their solubility in water at the laboratory temperature of 70 °F (21.1 °C) with Multiflash advanced thermodynamic modeling software that uses Cubic-Plus-Association (CPA) equation of state calculations (Fig. 4). Oxygen solubility shows a linear increase from 43.25 mg O_2_/L H_2_O at 1 atmosphere of pure oxygen pressure to 2723.29 mg O_2_/L H_2_O at 1000 psi, neglecting compressibility effects. At ambient atmospheric air pressure (14.7 psi), the equilibrium concentration of oxygen in water due to partial pressure is approximately 9.2 mg O_2_/L H_2_O (pO_2_ = 3.08 psia).

The amount of O_2_ dissolved in water can vary with changes in atmospheric composition and pressure, and by solubility modification due to temperature, salinity, and salt type^38,39^. We use changes in these conditions to vary dissolved O_2_ in water (aqueous solutions) and measure the corresponding changes in NMR fluid relaxation time.

### Pressurization of a Berea sandstone core with O_2_

We evaluated effect of dissolved oxygen on T2LM in porous media using a Berea sandstone core (length = 2 in., diameter ≈ 0.75 in.). The core was fully saturated in a vacuum chamber with DI water prior to the experiment and put in the NMR pressure vessel. A minimal head space of a few mm of DI water was added to the top of the core to prevent gas drying when pressurized. NMR measurements were made at ambient pressure (11.9 psi, pO_2_ = 2.50 psia), at a vacuum of − 24 in. Hg (pO_2_ = 0.02 psia), and at an oxygen gauge pressure of 10 psig (pO_2_ = 21.90 psia). Due to the multi-exponential decay of the NMR magnetization and the difference in relaxation times of the bulk fluid and pore fluid, pore fluid relaxation was distinguishable from relaxation of the free fluid head, and was used to evaluate pore fluid changes due to pO_2_ effects.

## Supplementary Information

Supplementary Information.
