# Supplementary material for: Quantification of dissolved O2 in bulk aqueous solutions and porous media using NMR relaxometry
Source: Sci Rep. 2021 Jan 11;11:290. doi: 10.1038/s41598-020-79441-5 (PMC7801431; doi:10.1038/s41598-020-79441-5)
Supplement: Supplementary file 1 — Supplementary Information. [file 41598_2020_79441_MOESM1_ESM.pdf]

SUPPORTING INFORMATION

Quantification of Dissolved O_2 in Bulk Aqueous Solutions and Porous Media Using NMR Relaxometry

Kurt Livo[1]*, klivo@mymail.mines.edu

Manika Prasad[1], mprasad@mines.edu

Trent R. Graham[2], Trenton.graham@pnnl.gov

[1]Center for Rock & Fluid Multiphysics, Colorado School of Mines, Golden, Colorado 80401, United States

[2]Pacific Northwest National Laboratory, Richland, Washington 99352, United States

CORRESPONDING AUTHOR

*Kurt Livo: klivo@mymail.mines.edu

Table S1. NMR Logarithmic Mean of Transverse Relaxation (T2LM) values and changes in T2LM for each pressure step during oxygen (O_2) injection in DI water in this study. NMR values in bold are repeat measurements during up and down pressure steps and show a lack of hysteresis in the values. The grey colored row marks ambient PT conditions to compare with other PT NMR data.

Gauge Pressure	Absolute Oxygen Partial Pressure, pO_2 (psia)	T2LM (ms)	Differential T2LM Value from Prior Pressure Step, ΔT2LM (ms)	Percentage Change in T2LM from Vacuumed State, ΔT2LM %	Dissolved O_2 in Solution (mg/L)	Molar % O_2 in Solution
24.0 in. Hg Vacuum	0.02	3388.18			0.07	0.0000 %
18.0 in. Hg Vacuum	0.64	3129.06	663.61	0.00 %	1.91	0.0001 %
0 psi No Vacuum	2.50	2465.45	0.00	21.21 %	7.45	0.0005 %
Oxygen (O_2) Pressurization						
100 psi Up	111.90	**293.58**	-2835.47	90.62 %	329.87	0.0223 %
200 psi Up	211.90	159.00	-134.58	94.92 %	618.32	0.0419 %
300 psi Up	311.90	109.03	-49.96	96.52 %	900.77	0.0614 %
400 psi Up	411.90	**84.62**	-24.41	97.30 %	1177.21	0.0807 %
500 psi Up	511.90	68.98	-15.65	97.80 %	1447.66	0.0998 %
600 psi Up	611.90	58.11	-10.87	98.14 %	1712.10	0.1186 %
700 psi Up	711.90	**50.55**	-7.55	98.38 %	1970.55	0.1373 %
800 psi Up	811.90	44.83	-5.72	98.57 %	2223.00	0.1558 %
900 psi Up	911.90	40.27	-4.56	98.71 %	2469.44	0.1741 %
1000 psi Up	1011.90	36.65	-3.62	98.83 %	2709.89	0.1921 %
Oxygen (O_2) Depressurization						
700 psi Down	711.90	**50.57**	50.57	98.38 %	1970.55	0.1373 %
400 psi Down	411.90	**84.51**	33.94	97.30 %	1177.21	0.0807 %
100 psi Down	111.90	**289.90**	205.40	90.74 %	329.87	0.0223 %
80 psi Down	91.90	350.14	60.24	88.81 %	271.46	0.0183 %
60 psi Down	71.90	439.40	89.26	85.96 %	212.81	0.0143 %
40 psi Down	51.90	587.51	148.11	81.22 %	153.93	0.0104 %
20 psi Down	31.90	911.80	324.29	70.86 %	94.80	0.0064 %
18.0 in. Hg Vacuum Down	3.06	2238.09	1326.29	28.47 %	9.11	0.0006 %

Table S2. NMR Logarithmic Mean of Transverse Relaxation (T2LM) values for each pressure step in water-saturated Berea sandstone and surrounding bulk fluid during oxygen (O_2) injection.

Gauge Pressure	Absolute Oxygen Partial Pressure (psia)	Bulk Fluid T2LM (ms)	Large Pores T2LM (ms)	Small Pores T2LM (ms)
Ambient State	2.50	2407.02	263.90	52.72
24.0 in. Hg Vacuum	0.02	2872.37	316.51	71.71
10 psi Oxygen Pressure	21.90	1085.76	203.56	47.79
